# Spectral domain optical coherence tomography findings of acute branch retinal artery occlusion from calcific embolus

**DOI:** 10.4103/0301-4738.71703

**Published:** 2010

**Authors:** Vinay A Shah, Billi Wallace, Nelson R Sabates

**Affiliations:** Department of Ophthalmology, University of Missouri Kansas City School of Medicine and Vision Research Center at Truman Medical Center, Kansas City, MO

**Keywords:** Artery, optical coherence tomography, retina, retinal artery occlusion, spectral domain

## Abstract

An 82-year-old female presented with sudden painless decrease in vision in the right eye after awakening. She could see the “superior half” of her vision from the right eye only. On examination, best-corrected vision was 20/300 in the right eye and 20/30 in the left eye. The fundus in the right eye revealed recent superotemporal branch retinal artery occlusion (BRAO) with calcified plaque at the disc. Spectral domain optical coherence tomography (OCT) (OTI Ophthalmic Technologies, Inc.), revealed hyperreflectivity and increased thickness of the inner retinal layers of the superior compared to the inferior retina. Imaging at the optic disc revealed the blocked artery containing a highly reflective material. The high reflectivity of the material and underlying optical shadowing could be characterized as calcific emboli.

Branch retinal artery occlusion (BRAO) presents as an acute painless loss of visual field in the distribution of the occluded artery. Commonly, BRAO occurs secondary to an embolus. Emboli typically originate within vessels upstream where they dislodge and travel within the circulatory system to ultimately become lodged downstream in a vessel with a smaller lumen. The most common are cholesterol emboli from aorto-carotid atheromatous plaques, platelet-fibrin emboli from thrombotic disease, and calcific emboli from cardiac valvular disease. Emboli are identified in ≈30% of the cases. Optical coherence tomography (OCT) findings of BRAO have been described in the literature using stratus OCT.[1,2]

Spectral domain OCT has been recently introduced for clinical use. This system allows a faster acquisition time than the conventional time domain OCT, thus allowing larger number of images to be acquired. This increased density of A-scans within B-scans, result in higher resolution OCT scans.[3,4] We present spectral domain OCT findings in a patient with BRAO secondary to a calcific embolus.

## Case Report

**Figure 1:**
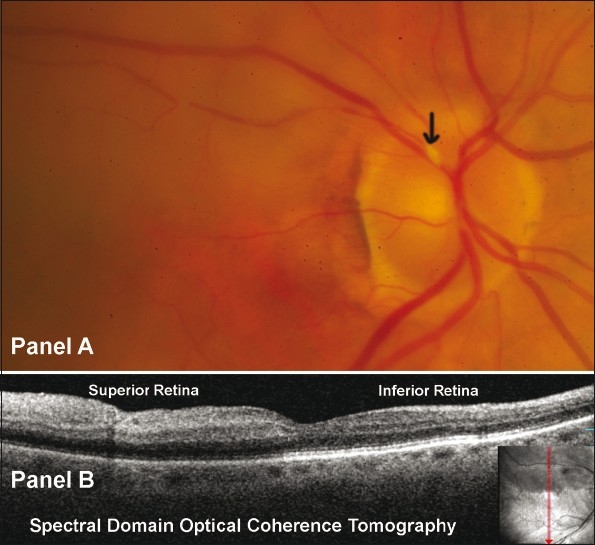
(A) Right Eye fundus photo revealing whitening of the superior retina with a calcified plaque at the disc within the supero temporal artery (arrow); (B) Spectral domain optical coherence tomography OCT/SLO, OTI Ophthalmic Technologies Inc, Ontario, Canada) revealed hyperreflectivity and increased thickness of the inner retinal layers in the superior compared to inferior retina. Note the decreased reflectivity of the outer retinal layers (including retinal pigment epithelial layer) in the superior retina as compared to inferior retina probably due to optical shadowing.

An 82-year-old female was admitted to the “stroke unit” with sudden painless decrease in vision in the right eye after awakening from her afternoon nap the previous day. Patient denied any headache, jaw pain, recent weight loss, amaurotic episodes, or any other neurologic symptoms. She still could see the “superior half” of her vision from the right eye. Past medical history was significant for hypertension, hypothyroid, aortic and mitral valve calcification, cerebrovascular accidents, and atrial fibrillation for which she was taking coumadin. Past ocular history was significant for blepharospasm. On examination, best-corrected visual acuity (BCVA) (Snellen) was 20/300 in the right eye and 20/30 in the left eye. Confrontation visual field revealed a loss of inferior visual field in the right eye and was full in the left eye. There was a relative afferent pupillary defect in the right eye. Anterior segment examination was unremarkable in both eyes. Dilated fundus examination in the left eye was remarkable for myelinated nerve fiber around the disc with a cup-to-disc ratio of 0.20. Fundus examination in the right eye was suggestive of recent superotemporal BRAO with calcified plaque at the disc within the superotemporal artery [Fig. 1A]. Fluorescein angiography confirmed the superotemporal BRAO.

**Figure 2:**
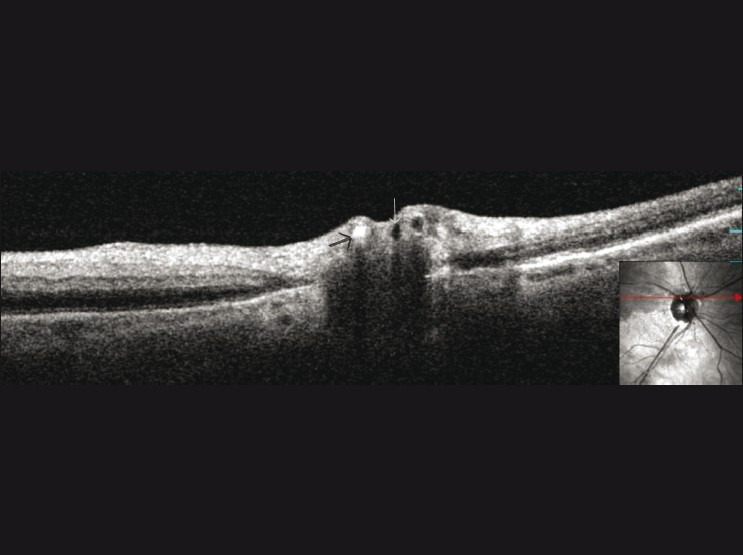
Spectral domain optical coherence tomography through optic disc revealed blocked artery with highly reflective material and optical shadowing likely due to calcified embolus (black arrow). There are adjacent vessels without blockage (white arrow)

Spectral Domain OCT (OCT/SLO, OTI Ophthalmic Technologies Inc, Ontario, Canada) revealed hyperreflectivity and increased thickness of inner retinal layers, and decreased reflectivity in the outer retinal layers, in the superior compared to inferior retina [Fig. 1B]. On optic disc imaging, the spectral domain OCT showed the calcified plaque as an area of high reflectivity within the lumen of the blocked artery with underlying optical shadowing [Fig. 2]. Due to delayed presentation (>24 hours) no treatment was offered. An urgent erythrocyte sedimentation rate, C-reactive protein, and complete blood count were normal. A repeat echocardiogram and carotid doppler ultrasound were recommended.

## Discussion

Retinal artery embolus commonly occurs from cholesterol (ulcerated carotid artery plaque), platelet fibrin (ulcerated vessel wall thrombus), and calcific (cardiac valve disease). Given the patient’s history of calcification of the mitral and aortic valves and the white color of the embolus, a calcific embolus from the heart is most likely.

OCT is increasingly being used in various retinal disorders due to its user friendliness and high resolution imaging capabilities. Spectral domain OCT allows *in vivo* retinal cross sectioning with resolution up to 5 microns.[4] In this case, a vertical scan through the macular area revealed hyperreflectivity and thickness of the nerve fiber and inner retinal layers in the superior ischemic retina compared to inferior unaffected retina in the acute phase. These acute OCT changes are similar to conventional Stratus OCT findings in arterial occlusions.[1,2,5] Decreased reflectivity of the outer retinal layers in the superior half, probably appears to be secondary to the optical shadowing effect. This is the first report of spectral domain OCT imaging visualizing the intraarterial embolus and initial report of spectral domain OCT findings of BRAO occlusion. The high reflectivitity and underlying optical shadowing may be a characteristic of the calcific embolus on spectral domain OCT. The ability to classify the type of embolus using spectral domain OCT in addition to the characteristics of the embolus on fundus examination may provide us with the possible etiology and/or sight of origin of the embolus in the patient. This may allow early diagnosis and treatment of the underlying etiology, which may be reduced mortality and morbidity in these patients. However, a larger series of eyes with OCT findings of emboli is required to establish these findings.
